# Abdominal surgery in patients with essential thrombocythemia: A case report and systematic review of literature

**DOI:** 10.1097/MD.0000000000008856

**Published:** 2017-11-27

**Authors:** Yi Zhu, HongGang Jiang, ZhiHeng Chen, BoHao Lu, JiaMing Wu

**Affiliations:** Department of Gastroenterological Surgery, First Hospital of Jiaxing, Jiaxing, Zhejiang, China.

**Keywords:** abdominal surgery, essential thrombocythemia, essential thrombocytosis

## Abstract

**Introduction:**

Essential thrombocythemia/thrombocytosis (ET) is characterized by increased bleeding and thrombosis risk during the perioperative period. We report the case of a woman with ET and sigmoid colon cancer, in whom the postoperative course was complicated by anastomotic bleeding. A systematic review was conducted to seek guidance for the management of such patient in the perioperative period.

**Methods:**

A systematic literature review was conducted using EMBASE, Medline, and PubMed databases to detect relevant English language articles. Published studies with full-text articles were included. Two authors independently searched and extracted the data. Any differences were resolved by consensus. Studies on abdominal surgery were manually retrieved.

**Results:**

Four case reports (including our case report) that described abdominal surgery in patients with ET were included. All patients were females, with a mean age of 47 years. Laparoscopic surgery was performed in 2 patients, and open surgery was performed in the other 2 patients. Two patients had postoperative bleeding that occurred on the first postoperative day. There was one case of pseudohyperkalemia after surgery and one case of Budd-Chiari syndrome caused by hepatic vein thrombosis. No guidelines for patients with ET undergoing abdominal surgery were found.

**Conclusion:**

In conclusion, there are currently no definitive guidelines for the perioperative management of patients with ET. Furthermore, there are few reports of ET in patients with malignancy undergoing surgery. Further studies in this unique group of patients are required.

## 1 Introduction

Essential thrombocythemia/thrombocytosis (ET) is a myeloproliferative disorder, in which sustained megakaryocyte proliferation leads to an increase in circulating platelets. It is characterized by bleeding and thrombosis risk during the perioperative period. However, the perioperative management of patients with ET remains unclear, especially in the context of abdominal malignancy.

We report a case of a female with ET and sigmoid colon cancer, in whom the postoperative course was complicated by anastomotic bleeding. A systematic review was conducted to search for other similar patients and seek guidance for the management of such patients in the perioperative period.

## 2 Case report

A 61-year-old female presented to our hospital with 1 month of rectal bleeding. Her medical history included hypertension and chronic schistosomiasis. She lived in an endemic area of *Schistosoma japonicum*, and has not traveled abroad. She reported no recent sexual contacts or recreational drug use. She was a nonsmoker and nondrinker.

On examination, the patient was afebrile. Her heart rate was 75 beats/min, blood pressure was 138/72 mm Hg, respiratory rate was 19 breaths/min, and oxygen saturation was 99% on air. There were no lesions in the oropharynx, and there was no lymphadenopathy. The lungs were clear and her heart rate was regular without murmur. There was no abdominal organomegaly. Her digital rectal examination was normal.

Laboratory investigations revealed a normal leukocyte count (5.07 × 10^9^ L^−1^, N: 4–10 × 10^9^ L^−1^), but her platelet count was significantly elevated (915 × 10^9^ L^−1^, N: 100–300 × 10^9^ L^−1^). Coagulation function was normal. Liver and renal function, carcinoembryonic antigen, and carbohydrate antigen 199 were within the normal range.

Colonoscopy revealed an ulcerating mass in the sigmoid colon. The pathological diagnosis was adenocarcinoma. Computed tomography (CT) of the chest, abdomen, and pelvis were negative for metastases. Abdominal MRI revealed splenomegaly. Bone marrow biopsy revealed megakaryocyte lineage proliferation. There was no significant proliferation or left shift in granulocytes and red cell lines. She had a mutation in clonal marker JAK2V617F.

Plateletpheresis was performed 2 days before surgery. This reduced her platelet count to 444 × 109 L^−1^. The laparoscopic approach was selected to decrease the size of the incision, decrease intraoperative bleeding, lower overall complications, and enable faster recovery.^[1–3]^ Following resection of the lesion in the lower part of the sigmoid colon, intestinal continuity was reestablished with a circular stapler, and interrupted sutures were used for reinforcement and hemostasis. Intraoperative bleeding totaled to approximately 50 mL. Anticoagulants for the prevention of postoperative thrombosis were not used due to concerns regarding postoperative bleeding.

Postoperatively, blood pressure remained stable and did not become elevated. Anastomotic bleeding occurred on postoperative day one. The initial amount of bleeding was approximately 50 mL. Platelet count at this stage was 801 × 109 L^−1^. Dilute noradrenaline was used as a retention enema, and 0.5 U of hemocoagulase was injected. Conventional postoperative anticoagulation was not started due to bleeding. Bleeding was controlled within 3 days after surgery. The total amount of bleeding was approximately 400 mL. Liquid feeding was allowed on the fifth postoperative day. The patient was discharged on postoperative day 13 with no complications. The pathological stage of the cancer was T4aN1M0.

The patient did not receive postoperative chemotherapy or ET related therapy. At postoperative 7 months, CT examination revealed pulmonary and liver metastases. She was referred to medical oncology for palliative chemotherapy.

## 3 Methods

A systematic literature review was conducted using EMBASE, Medline, and PubMed databases to search for relevant English language articles published up to March 2017. The following search terms were used: “essential thrombocythemia” OR “primary thrombocytosis” AND “surgery” OR “surgical” OR “operation” OR “operative.” All eligible studies were retrieved, and the bibliographies were checked for other relevant publications. Published studies with full-text articles were included. Two authors independently searched and extracted the data. Any differences were resolved by consensus. Studies on abdominal surgery were manually retrieved. The ethical approval was not necessary, because this is a retrospective case report. And the informed consent of the patient was given.

## 4 Results

A total of 65 studies were initially found. Based on the inclusion criteria, 8 studies were selected for review. Among these 8 studies, 4 studies did not meet the inclusion criteria; while the remaining 4 studies (including this case report) described abdominal surgery in patients with ET and were included (Table 1). All patients were female, and the mean age of these patients was 47 years. Laparoscopic surgery was performed in 2 patients, and open surgery was performed in the other 2 patients. Two patients experienced postoperative bleeding that occurred on the first postoperative day. Anticoagulants were terminated or were not commenced in cases of bleeding (Table 2). There was 1 case of pseudohyperkalemia after surgery and 1 case of Budd-Chiari syndrome caused by hepatic vein thrombosis.

**Table 1:**
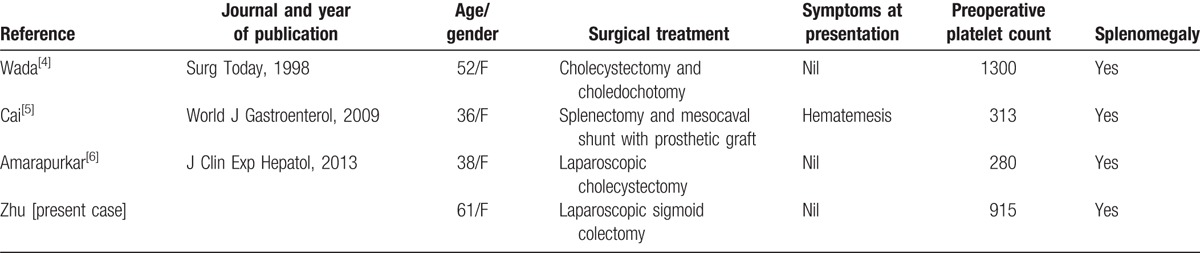
Description and summary of eligible studies.

**Table 2:**
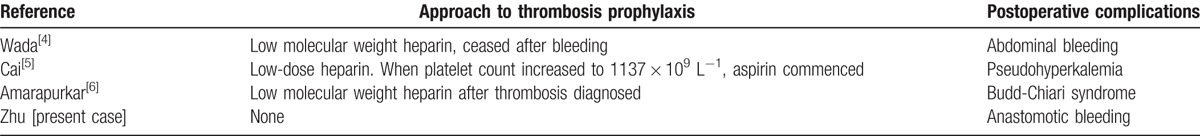
Postoperative information.

## 5 Discussion

This case report describes a patient with colonic malignancy and ET. From the systematic review of literature, only 3 studies on patients undergoing abdominal surgery with ET were identified. No clear guidelines for the perioperative management of patients with ET undergoing abdominal surgery were identified.

ET is a rare disease that has an incidence of 2.5/100,000/year.^[7]^ The risk of postoperative bleeding and thrombosis are increased for patients with ET.^[8]^ Furthermore, the treatment of ET in colorectal cancer is complex, and requires a multidisciplinary approach that involves colorectal surgeons, hematologists, anesthesiologists, and pharmacists.

Nand et al found that the rate of secondary thrombocytosis (ST) associated with malignancy may be as high as 30% to 60%.^[9]^ Griesshammer et al analyzed 732 patients with thrombocytosis, and revealed that the proportion of patients with ST caused by malignancy is far higher than patients with ET.^[10]^ Thrombosis is a major hazard in ET.^[11]^ Thrombosis appears to occur more frequently in ET than in ST.^[10]^ Furthermore, the surgery itself enhances perioperative thrombotic risk up to 5-fold, and it would be expected that a concomitant thrombocytosis would further amplify this risk.^[12]^

Major complications such as pulmonary embolism have been reported after abdominal or cardiac surgery in patients with ET.^[13,14]^ Therefore, caution during preoperative evaluation and treatment to reduce platelet counts are considered to be essential before surgery.^[15–18]^ We chose plateletpheresis to quickly decrease the platelet count, since this has been successfully applied in coronary artery bypass grafting.^[19]^

The incidence of anastomotic bleeding after colorectal surgery is low. From our previous studies, chronic schistosomiasis, similar to our patient, did not increase the risk of postoperative anastomotic bleeding in colorectal cancer.^[20]^ Randi ML et al revealed that hemorrhage after surgical procedures was not uncommon in patients with ET.^[21]^ Therefore, postoperative anastomotic bleeding is likely to be increased in patients with ET.

This systematic review was limited by available literature. No clear guidelines for the management of patients undergoing abdominal surgery were available to base the management of our patient. Patients with abdominal malignancy and ET are a rare group of patients, since these patients have increased thrombotic risk from the malignancy itself and increased risk of disorders of coagulation function from the ET. Our management included plateletpheresis, which was based on work in cardiac surgical patients. Whether this can be generalized to patients such as the patient described in this study remains unclear.

In conclusion, there are no definitive guidelines for the perioperative management of patients with ET. Furthermore, there are only few reports on ET in patients undergoing abdominal surgery, and both thrombosis and bleeding seem to be important complications. The decrease in platelet count appears to be important preoperatively, and plateletpheresis can rapidly achieve this. Further studies in this unique group of patients are required.

## References

[1] Kim SH Park IJ Joh YG Laparoscopic resection for rectal cancer: a prospective analysis of thirty-month follow-up outcomes in 312 patients. Surg Endosc 2006;20:1197–202. 16865622 10.1007/s00464-005-0599-2

[2] Yong L Deane M Monsoon JPT Systematic review of laparoscopic surgery for colorectal malignancy. Surg Endosc 2001;15:1431–9. 11965460 10.1007/s004640090131

[3] Yan SL Xu ZB Chi P Comparing the influencing factors of anastomotic bleeding in rectal carcinoma resection between laparoscopic and open radical approaches. Zhongua Wei Chang Wai Ke Za Zhi 2007;10:157–9. 17380458

[4] Wada Y Ryo J Sarumaru S Surgery for cholecystocholedocholithiasis in a patient with asymptomatic essential thrombocythemia: report of a case. Surg Today 1998;28:1073–7. 9786583 10.1007/BF02483965

[5] Cai XY Zhou W Hong DF A latent form of essential thrombocythemia presenting as portal cavernoma. World J Gastroenterol 2009;15:5368–70. 19908349 10.3748/wjg.15.5368 PMC2776868

[6] Amarapurkar PD Parekh SJ Sundeep P Budd-Chiari syndrome following laparoscopic cholecystectomy. J Clin Exp Hepatol 2013;3:256–9. 25755508 10.1016/j.jceh.2013.07.001 PMC4216828

[7] Hehlmann R Jahn M Baumann B Essential thrombocythemia. Clinical characteristics and course of 61 cases. Cancer 1988;61:2487–96. 3365670 10.1002/1097-0142(19880615)61:12<2487::aid-cncr2820611217>3.0.co;2-t

[8] Levine SP Greer JP Thrombocytosis. Wintrobe's Clinical Hematology 11th ed. Philadelphia, PA: Lippincott Williams & Wilkins; 2006 1591–600.

[9] Nand S Messmore H Hemostasis in malignancy. Am J Hematol 1990;35:45–55. 2202206 10.1002/ajh.2830350111

[10] Griesshammer M Bangerter M Sauer T Aetiology and clinical significance of thrombocytosis: analysis of 732 patients with an elevated platelet count. J Intern Med 1999;245:295–300. 10205592 10.1046/j.1365-2796.1999.00452.x

[11] Fenaux P Simon M Caulier MT Clinical course of essential thrombocythemia in 147 cases. Cancer 1990;66:549–56. 2364366 10.1002/1097-0142(19900801)66:3<549::aid-cncr2820660324>3.0.co;2-6

[12] Harrison CN Bareford D Butt N Guideline for investigation and management of adults and children presenting with a thrombocytosis. Br J Haematol 2010;149:352–75. 20331456 10.1111/j.1365-2141.2010.08122.x

[13] Schölzel BE Endeman H Dewilde W Cardiac surgery in a patient with essential thrombocythemia: a case report. Neth Heart J 2010;18:378–80. 20730008 10.1007/BF03091797 PMC2922787

[14] Nishikawa H Fujimoto K Koide Y Postoperative pulmonary thromboembolism in a patient with essential thrombocythemia. Masui 2010;59:738–9. 20560378

[15] Kiro K Ganjoo P Saigal D Incidental thrombocytosis: Should it concern the anesthesiologist? J Anaesthesiol Clin Pharmacol 2014;30:281–3. 24803776 10.4103/0970-9185.130102 PMC4009658

[16] Edlich RF Long WB III Cochran AA Management of femoral fracture in a patient with essential thrombocythemia treated with plateletpheresis and intramedullary rod fixation, followed by hydroxyurea: a case report. Am J Emerg Med 2008;26:636.e1–3. 10.1016/j.ajem.2007.09.025 18534315

[17] Ahmed K Vohra HA Milne A Aortic valve replacement in a young patient with essential thrombocytosis. J Cardiothorac Surg 2008;3:5. 18234096 10.1186/1749-8090-3-5 PMC2246126

[18] Sugimoto T Kitade T Mimura T Infrainguinal bypass surgery for chronic arterial occlusive disease associated with essential thrombocythemia: report of a case. Surg Today 2004;34:632–5. 15221564 10.1007/s00595-004-2759-8

[19] Das SS Bose S Chatterjee S Thrombocytapheresis: managing essential thrombocythemia in a surgical patient. Ann Thorac Surg 2011;92:e5–6. 21718822 10.1016/j.athoracsur.2011.02.050

[20] Yi Z Hong-Gang J Zhi-Heng C Short-term efficacy of laparoscopic treatment for colorectal cancer in patients with schistosomiasis japonica. Gastroenterol Res Pract 2016;2016:8357025. 27843449 10.1155/2016/8357025 PMC5098079

[21] Randi ML Stocco F Rossi C Thrombosis and hemorrhage in thrombocytosis: evaluation of a large cohort of patients (357 cases). J Med 1991;22:213–23. 1787383

